# Systematic Investigation of Biocompatible Cationic Polymeric Nucleic Acid Carriers for Immunotherapy of Hepatocellular Carcinoma

**DOI:** 10.3390/cancers14010085

**Published:** 2021-12-24

**Authors:** Mingsheng Chen, Hao Wang, Hongying Guo, Ying Zhang, Liang Chen

**Affiliations:** 1 Shanghai Public Health Clinic Center, Fudan University, Shanghai 201508, China; chenmingsheng@shphc.org.cn (M.C.); wanghao@shphc.org.cn (H.W.); guohongying@shphc.org.cn (H.G.); 2 School of Environmental and Chemical Engineering, Jiangsu University of Science and Technology, Zhenjiang 212100, China

**Keywords:** biocompatible cationic polymers, hepatocellular carcinoma, gene intervention, immunotherapy

## Abstract

**Simple Summary:**

Immunotherapy, including adaptive and innate immunotherapy, exhibits promising future for the treatment of hepatocellular carcinoma. As a common tool for immunotherapy, the safe and efficient gene delivery turns to be especially important. Biocompatible polymers are a category of promising materials used in gene delivery, while there still lacks a comprehensive review article to discuss the updates on multiple disciplines covering biocompatible polymers, gene therapy, tumor immune microenvironment, and immunotherapy. This review is well-integrated with biocompatible polymers, nonviral gene therapy, and cancer immunotherapy. Our investigation will provide different perspective for the scientists focusing on the domains of biomaterials, gene therapy, and oncologists to move their research work forward.

**Abstract:**

Hepatocellular carcinoma (HCC) is the third-largest cause of cancer death worldwide, while immunotherapy is rapidly being developed to fight HCC with great potential. Nucleic acid drugs are the most important modulators in HCC immunotherapy. To boost the efficacy of therapeutics and amplify the efficiency of genetic materials, biocompatible polymers are commonly used. However, under the strong need of a summary for current developments of biocompatible polymeric nucleic acid carriers for immunotherapy of HCC, there is rare review article specific to this topic to our best knowledge. In this article, we will discuss the current progress of immunotherapy for HCC, biocompatible cationic polymers (BCPs) as nucleic acid carriers used (or potential) to fight HCC, the roles of biocompatible polymeric carriers for nucleic acid delivery, and nucleic acid delivery by biocompatible polymers for immunotherapy. At the end, we will conclude the review and discuss future perspectives. This article discusses biocompatible polymeric nucleic acid carriers for immunotherapy of HCC from multidiscipline perspectives and provides a new insight in this domain. We believe this review will be interesting to polymer chemists, pharmacists, clinic doctors, and PhD students in related disciplines.

## 1. Introduction

Hepatocellular carcinoma (HCC) is an aggressive malignancy with a poor prognosis and the third-largest cause of cancer death worldwide [1]. It has received tremendous attention due to the sixth most frequent type of solid tumor and the second leading cause of cancer-related mortality cross the world [2]. The incidence rate of HCC has increased significantly in the past and is predicted to rise to 22 million by the next decade [3]. Chronic infection with hepatitis B (HBV) or hepatitis C viruses (HCV) is considered as the key risk factor for HCC [4,5,6], and HCC accounts for 50–80% of being hepatitis B virus-related [7]. A few HBV factors, including HBV X protein (HBx) and pre-S2/S gene, have been implicated in the progression of HCC [8]. The HBx is regularly found in HBV-related HCC, and it is commonly recognized that HBx plays a major role in HBV-related hepatocarcinogenesis [9]. Additionally, alcohol consumption, metabolic syndrome [10], dietary toxins, and diabetes are also verified to be the relevant risk factors associated with HCC [11].

As to the prevention and treatment, HCC surveillance and early detection contribute to potentially curative treatment [12]. Currently, surgical resection, liver transplant, and ablation are employed by most hospitals as potential curative therapies after diagnosis, followed with chemotherapy. However, the 5-year survival rate of HCC is still much lower than 20% due to rapid tumor growth, tumor drug resistance, and a high incidence of tumor recurrence and metastasis [13]. To overcome these problems, extensive research on the new therapeutics of HCC has been conducted. Recently, tyrosine kinase inhibitors (TKIs) and immunotherapy have been dominant for the treatment of HCC in preclinic and clinic. TKIs are a kind of chemotherapy agents developed over the past decades, and there are continuous efforts to develop more potent TKIs to improve morbidity and mortality for HCC patients [14], while tremendous challenges stop further clinic application, such as drug resistance and nonspecific cytotoxicity [15]. The research of cancer immunotherapy has made significant progress in treating HCC recently, and immunotherapy shows great potential to fight tumors [16]. Successful examples of tumor immunotherapy include chimeric antigen receptor T cell (CAR-T), CAR-NK, and PD1/PDL1 checkpoint-blocking, which already are applied in the clinic and have verified unique advantages comparing to chemotherapy. However, more than 80% patients do not show durable responses from antibody-based immunotherapy [17]. The disappointing prognosis of sole immunotherapy is because of the harsh tumor microenvironment. Recently, gene intervention has played a crucial role to promote the current cancer immunotherapy [18]. (1) Direct gene delivery to active or silence the immune associated genes has become an essential option for cancer immunotherapy, such as siRNA for PDL1 or CTLA-4 silencing. Although there remain no siRNA drugs approved by the Food and Drug Administration (FDA) of United States for cancer therapy, they are steadily progressing and show bright perspectives in clinic trials. (2) Interventions of oncogenes to modulate the tumor immune microenvironment (TIME) can significantly promote the progress of PD1/PDL1-based immunotherapy, such as MYC gene modulation in HCC. (3) Interventions of metabolism-associated genes to modulate TIME is another tool to enhance tumor immunotherapy [19]. Therefore, gene therapy and immunotherapy sometimes do not have an obvious boundary, and there are too many integrations.

**Figure 1:**
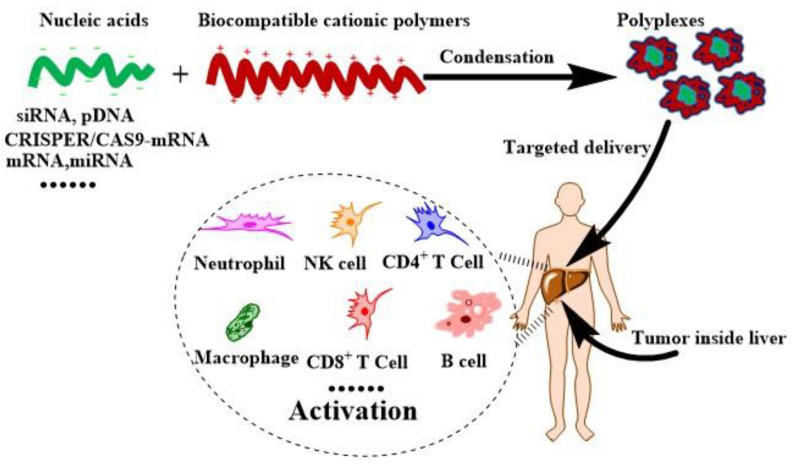
Scheme of nucleic acids delivered by BCPs.

For gene intervention, the right carriers are most critical once the sequences of nucleic acids are customed. The main problems of the current nonviral carriers are low transfect efficiency and high cytotoxicity. Nonbiodegradable polymers accumulated around the cells can trigger cytotoxicity and organ damage, which are not biocompatible with the physiological system [20]. Biocompatible cationic polymers (BCPs) have the nature of biodegradable, low cytotoxicity, no mutagenicity, and absence of carcinogenicity. On the other hand, the transfect efficiency of this kind of material can be enhanced through molecular modification, architecture design, and virus-mimicking. As nonviral carriers, BCPs, especially the cationic polysaccharides, polypeptides, and polyesters, have received more and more attention in preclinic and clinics (as shown in Figure 1) due to their safety and efficiency [21]. To achieve nucleic acids delivery, as well as other specific targets, the design and synthesis of biocompatible cationic polymeric carriers are developing rapidly. During gene delivery for immunotherapy, BCPs have the functions such as minimizing the unwanted immune response, amplifying the immune-response, and activating the immune cells [22]. Adaptive immunotherapy for HCC (including checkpoint blocking and CAR-T therapy), innate immunotherapy, and intervention of oncogenes/metabolism to modulate TIME show great demand with satisfied nonviral carriers for gene delivery. 

In this review, BCPs as nucleic acid carriers used or potential for HCC immunotherapy will be discussed, and especially, cationic polysaccharides, polypeptides, polyesters will be addressed. We will summarize the contributions of BCPs for nucleic acid protection, tumor targeting, transfect efficiency enhancement, and cytotoxicity minimization. BCPs are deeply involved in adaptive and innate immunotherapy; thus, their unique roles to enhance the current HCC therapy will be included. The progression and response to immunotherapy of HCC are decided by TIME in some cases. The modulation of TIME by oncogenes or metabolic interventions is verified as an effective option to promote the overall survival (OS) and will be discussed in detail here. In the meanwhile, the specific roles and encountered challenges during BCPs as gene carriers for HCC immunotherapy will be addressed. At the end, a summary followed by the proposal and the future perspectives as to the development of BCPs as gene carriers for HCC immunotherapy will be presented. 

Although BCPs have been extensively developed as nucleic acid carriers for cancer immunotherapy, most of materials were only evaluated in vitro and not stable physiological conditions. Consequently, a few BCPs are used for in vivo cancer immunotherapy, including HCC and other cancers. For immunotherapy, the HCC and other malignant tumors have many common characteristics. We believe BCPs used for immunotherapy in other tumors are good references for HCC, which can accelerate the applications of BCPs for HCC immunotherapy in the future, Therefore, some discussions of other tumors are also included in this review.

## 2. BCPs as Gene Carriers to Fight HCC

With low immunogenicity, low cost, large loading capacity, and high chemical versatility, cationic polymers have been extensively exploited for gene delivery. Various types of polymers have been specifically designed for gene delivery, such as intracellular barrier penetration, tissue response release, biocompatibility, and endosomal escape (as shown in Table 1). On one hand, biodegradable polymers, such as polyanhydrides, are not stable in vivo for the considered time and are difficult to incorporate positive charges for gene binding [23]. On the other hand, PAMAM, poly(ethylenimine) (PEI), and PDMAEMA are traditional gene carriers with good transfect efficiency, as well as unbearable cytotoxicity [24,25,26,27,28], which have a low chance of approval for clinic application. Herein, several classes of BCPs for gene delivery, including cationic polysaccharides, cationic polypeptides, and cationic polyesters [29,30], are listed to fight HCC (or with the potential for HCC therapy), which show the most promise. Table 1 has summarized the categories of polymers and their applications for nucleic acid delivery.

### 2.1. Cationic Polysaccharides

Nucleic acids need to overcome various physiological and intracellular barriers to reach the target tissues for successful delivery. Over the past decades, many efforts have been dedicated to crack down these diverse barriers efficiently. Cationic polysaccharides have shown high affinity to liver [52], and pullulan or chitosan-based polysaccharides are often used for the targeted gene therapy against HCC [53,54]. Large number of hydroxyls on the surface of polysaccharides contribute to excellent water solubility and biocompatibility. As gene carriers, some polysaccharides have the potential to interact with cells or tissues to promote the transfect efficiency. When gene interventions become a tool of directly or indirectly enhancing HCC immunotherapy, natural cationic polysaccharide will be an attractive material for delivery. The negatively charged surface of nucleic acids necessitates the cationization of polysaccharide. Spermine, spermidine, and 1,4-diaminobutane are naturally existing cations that are often used to modify polysaccharides. The conjugates of dextran–spermine are highly effective both in vitro and in vivo, and additional modification with increasing amounts of hydrophobic molecules resulted in higher gene transfection in vivo [55]. Toita and co-authors developed a gene delivery system capable of endosome disruption with a polysaccharide-based cationic nanogel to hydrolyze membrane phospholipids. The nanogel capsulated pDNA by hydrophobic and electrostatic interactions, and pDNA was effectively internalized into cells. Their data suggests that the polysaccharide-based cationic nanogel possesses membrane disruption ability when delivered into cells and stimulates the subsequent release of pDNA from the endosome to the cytoplasm [56]. Since the stability of gene delivery system is essential during the long circulation time in plasma, Yang and co-authors developed sodium alginate from four different polysaccharides to shield the positive charge. The shielded nanoparticles exhibited enhanced stability in vitro and in vivo [57]. Chitosan as a kind of natural cationic polysaccharide does not need further modification normally and serves as an excellent material for gene delivery mainly due to its acceptable positively charged surface, biodegradability, and biocompatibility [58]. 

Beside the natural polysaccharides, the synthesized cationic polysaccharides also are developed for gene therapy due to their flexibility of rational design. Kanamycin is an aminoglycoside antibiotic and used to prevent a wide variety of infections. As a model of aminoglycoside molecule, Kanamycin was investigated on the role of sugar in gene delivery. Obviously, Kanamycin as a small molecule cannot condense the pDNA into a nanostructure. Several Kanamycin were thus coupled with diethylene glycol diacrylate (DEGDA) to form sugar oligomers (the molecular weight is around 1 kDa). Basically, the gene transfection efficiency is corrected with the molecular weight of polycations. The efficiency of sugar oligomers is comparable with the low molecular weight chitosan (50–190 kDa based on viscosity) [31] when DEGDA was replaced by *N*,*N*′-methylenebisacrylamide (MBA). The molecular weight of the sugar oligomers was increased to 6.4 kDa, and the efficiency is 33-fold higher than chitosan [59]. Furthermore, Gentamicin was used as the aminoglycoside to replace Kanamycin, and the molecular weight further grows to 15 kDa, while the efficiency is higher than branched PEI (25kDa) in vitro. Additionally, the cationic polysaccharides can strongly inhibit the growth of cancer cells and bacteria [32]. Due to the stability and biological function of polysaccharides issues, glycopolypeptides via the living polymerization of glycosylated-L-lysine *N*-carboxyanhydrides (NCAs) were developed by the Deming group [60]. They prepared conformation-switchable glycopolypeptides with living polymerization. The glycopolypeptides are water-soluble and *α*-helical in aqueous solution. The oxidation of the side-chain thioether linkages resulted in disruption of the *α*-helical conformations without loss of water solubility. The modulation of conformation can mediate the spatiotemporal release of nucleic acids and probably contribute to the immunotherapy [61], which shows great potential for HCC immunotherapy with gene intervention.

### 2.2. Cationic Polypeptides

Polypeptides are a kind of biodegradable material composed of repeating amino acid units linked with peptide bonds and can be cleaved in vivo by specific enzymes [62]. Amphiphilic polypeptides can be assembled into nanostructure or by conjugating the metal solid core with hydrophilic polypeptides to form nanoparticles [33]. Similar to polysaccharides, natural peptides and synthetic polypeptides both are chosen for nucleic acid delivery. 

**Figure 2:**
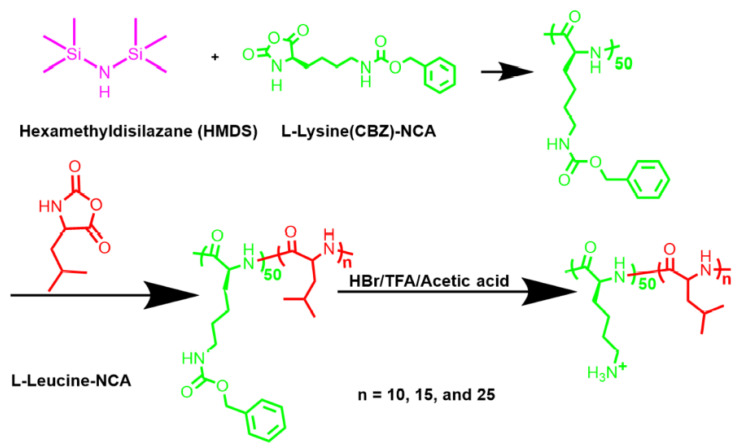
Synthesis route of controlled di-block amphiphilic poly(L-lysine)_50_-*block*-poly(L-leucine)_n_ ([36] *Polymers* **2018**, *10*, 379).

In the category of synthetic polypeptides, NCA-ring opening polymerization is gaining attention. With decades of development, NCA-ring opening has become a controlled method for the synthesis of polypeptides [63,64]. Higher structures, physical and chemical properties, topologies, and other parameters of polypeptides can be tailored via the design of synthesis. As gene carrier, the customized polypeptides derived a wide variety of biological functions that contributed to HCC gene/immunotherapy. Wang reported an efficient CRISPR/CAS9 delivery system based on *α*-helical polypeptide. Being assisted by the high membrane-penetrating ability of polypeptides, the nanoparticles achieved efficient cellular internalization and endosomal escape. The CRISPR/CAS9 loaded polypeptides could reach 47.3% gene editing in cells, 35% gene deletion in tissues, and >71% tumor inhibition, demonstrating an advantage over the existing conventional polycationic vectors [34]. Chen and co-authors prepared shell-stacked nanoparticles based on core-shell polypeptides. The disulfide cross-linked core maintains the stability of the nanoparticle and stops undesired premature release of therapeutics, while it can accelerate the cleavage of more disulfide bond and promote intracellular drug release. The resulted nanoparticles showed significant antitumor efficacy and nearly eradicated the tumor [35]. Due to the important role of architectures, tremendous efforts have been spent to synthesize architecture-controlled polypeptides. Baumgartner reported a polypeptide with spatially organized *α*-helices, which are grown from a high-density initiating group [65]. As shown in Figure 2, we used *1*,*1*,*1*,*3*,*3*,*3*-hexamethyldisilazane (HMDS) as the initiator to produce controlled block amphiphilic polypeptides [36]. With the growth of poly(L-leucine) chain, the *α*-helix became stronger, and the gene transfect efficiency was steadily modulated by the length of the hydrophobic chain. 

As discussed above, synthetic polypeptides have advantages compared to natural peptides with well-defined nanostructures and large-scale synthesis, while the latter also have their advantages and are used for gene delivery. Especially, the natural peptides sometimes have strong biological functions, such as an affinity to specific organs for targeted delivery. Liang and co-authors employed epidermal growth factor receptor (EGFR)-binding peptide amphiphile to construct ultra-stable self-assembling peptide nanovesicles. The resulted nanoparticles could efficiently encapsulate therapeutic siRNAs or labeled fluorescent cargo and exhibited excellent affinity for EGFR-positive cancer cells. Moreover, the nanoparticles could deliver more plasmid DNA to tumor sites and promote gene expression [37]. Specific sequences of peptides have specific functions to enhance gene delivery. For example, the RGD-incorporated peptides have high efficiency in penetrating cancer cells. Mason and co-authors demonstrated that histidine-rich amphipathic peptide have significant DNA transfection capabilities. They found that the helix length and positioning of the histidine residues play important roles to obtain an optimal resistance to serum effects and DNA escape from the endosome [38]. Virus-derived membrane-permeable arginine-rich peptides have been shown to possess a transfect ability in cell lines [66,67]. Protamine is a highly positively charged peptide isolated from salmon sperm protein with a molecular weight of ~4.5 kDa. As early as 1997, it was already used for gene delivery [68]. 

### 2.3. Cationic Polyesters

Polyesters are the polymers linked by ester bonds and metabolized through hydrolysis or enzymatic digestion at physiological conditions. Currently, aliphatic polyesters, polyphosphoesters, and poly(*β*-amino ester) are most commonly used for gene delivery.

Aliphatic polyesters, which include polycaprolactone, polylactide, polyglycolide, and their derivatives, have been utilized in a number of FDA-approved products for gene delivery. Novel synthetic strategies to generate functionalized polyesters are strongly desired to improve their physical properties toward their application on gene delivery. Rapid synthesis of a polyester library is a robust approach to find a carrier for efficacious siRNA delivery. The Siegwart group screened functional polyesters for selective siRNA delivery to cancer cells [69]. Hao and co-authors reported an approach to rapidly synthesize a library with of >130 lipocationic polyesters directly from functional monomers. The screened polyesters were highly effective for siRNA delivery [39]. Poly (D,L-lactide-*co*-glycolide) (PLA) is a kind of biodegradable material and got approved for clinic application by the FDA. PLA based-nanoparticles as drug carriers have been extensively developed for HCC therapy [40]. 

Polyphosphoesters are another type of biocompatible material for therapeutics delivery [41]. Polyphosphoesters are characterized with fast degradation under physiological conditions. The architectures of polyphosphoesters can be versatile by controlled ring-opening polymerization [42]. Wang group designed and synthesized poly(2-aminoethyl ethylene phosphate)-based copolymers for siRNA delivery. The copolymers can condense siRNA into well-defined nanoparticles with effective internalization and subsequent siRNA release inside cells, resulting in efficient gene knockdown activities. Cationic micelles made from biocompatible and biodegradable polyphosphoesters are promising for siRNA delivery [43]. A new biodegradable polyphosphoester was synthesized and investigated for gene delivery by the Leong group. With the incorporation of a positive charge on its backbone and a lipophilic cholesterol on the side chain, the polyphosphoesters could bind and protect plasmid DNA from nuclease digestion. In vivo studies showed a gene expression in muscle increasing within 3 months. The two parameters of backbone charge density and the side chain lipophilicity can be modulated through copolymerization and monomer variation to optimize the transfection efficiency [44]. Galactosylated polyphosphoramidates with different ligand substitution degrees were prepared as hepatocyte-targeted gene carriers as another research area from the Leong group. The affinity of nanoparticles to galactose-recognizing lectin increases with the degree of galactose substitution and transfection efficiency mediated by ternary nanoparticles prepared with 6.5% galactose were significantly higher than the nanoparticles without galactose in hepatocytes at low N/P ratios [45]. 

Among the biodegradable nonviral gene vectors, poly(beta-amino ester)s (PBAEs) have risen as leading gene carriers that have been used for multiple applications in vitro and in vivo [46]. PBAEs were first synthesized in 1983 and developed for gene therapy in 2000. Due to their excellent properties, PBAEs were amply explored to generate effective gene vectors in both in vitro and in vivo studies. Since very small changes in the structure of PBAEs can cause impressive impacts on the transfection efficiency, the rational design of PBAEs is a major focus aiming to achieve high transfection efficiencies [47]. The Langer lab prepared PBAEs with two distinct structures. Twelve unique versions of each structure were synthesized by modulating amine/diacrylate stoichiometric ratios, resulting in PBAEs with either amine or acrylate end groups. Through the optimization of molecular weight, chain end group, and polymer/DNA ratio, these PBAEs successfully mediated gene delivery more efficiently than both PEI and Lipofectamine 2000 in vitro [48]. Zhou described the synthesis of a series of poly(amine-*co*-ester) terpolymers with high molecular weight and low charge density and showed efficient gene delivery. The poly(amine-*co*-ester)s were synthesized via enzyme-catalyzed copolymerization and tuned hydrophobicity. The targeted delivery of the proapoptotic TRAIL gene to tumor xenografts by one of the poly(amine-*co*-ester)s contributed to the significant inhibition of tumor growth, with tolerant toxicity both in vitro and in vivo [49]. The Wang group reported the design and synthesis of highly branched PBAEs via the Michael addition approach and evaluated the transfect potential. The branched structure can significantly enhance the transfection efficiency of PBAEs in vitro and in vivo [50].

Biocompatible polyesters are one of most promise materials for HCC for cancer therapy, and some of them got approved by the FDA, indicating their safety and suitability as therapeutic vectors. With the conjugation of cationic molecules, many biocompatible polyesters have been conducted preclinic or clinic studies for gene therapy. HCC immunotherapy with gene intervention is in great demand, and biocompatible cationic polyesters as gene carriers will continue to be developed.

## 3. The Specific Roles of Biocompatible Cationic Polymeric Carriers for Gene Delivery

Although the cancer cells are killed by various active agents, the polymeric carriers also play a key role to enhance the efficacy. Biocompatible polymers are used as gene carriers for the therapeutics of siRNAs, miRNAs, plasmid DNA, and mRNA. In various nucleic acids formulations, polymers all share the most common and important functions, including protecting the nucleic acids, tumor targeting, and enhancing the transfect efficiency. Such functions are essentially needed by the genetic materials for HCC immunotherapy, as discussed below.

### 3.1. Protecting the Nucleic Acids

Exogenous nucleic acids often are degraded by all kinds of enzymes in plasma or eliminated by the immune system when they are injected without protection. Therefore, to protect the nucleic acids before they work at the liver is the essential goal for gene carriers to fight HCC, and cationic polymers are commonly selected to protect nucleic acids from premature degradation [51]. Guo and co-authors used PEI-based gene carriers to silence PBOV1 to validate the key oncogene which greatly promotes HCC proliferation [70]. We developed a biocompatible polysaccharide hyperbranched poly(kanamycin-MBA) (HPKM) to protect plasmid DNA [55], which displays low cytotoxicity while a promising transfect efficiency. The therapeutic DNAs were condensed into well-defined nanoparticles, preventing the degradation from enzymes. Of course, the above efforts are just specific cases for gene protection. Herein, we will summarize the key points as to the protection of nucleic acids by polymers as the following.

(a) Protecting against enzyme attack

Therapeutic RNA and DNA are sensitive to various enzymes in the body, and gene materials must be protected by carriers for effectively therapy. Since varied materials often have diversity effects, carriers commonly are evaluated the by DNA or RNA condensation with agar gel electrophoresis (AGE) [55]. Since the ability of nucleases to perform their catalytic functions depends on the sequence and architectural properties of target DNA substrates, Keum and Bermudez developed an approach to enhance the resistance of DNA nanostructures to enzymatic digestion [71], which prepared several DNA tetrahedra with different sizes and shapes. Many DNA structures need a high ionic strength to maintain their integrity and can be degraded quickly by nucleases. Agarwal and co-authors packed couple of different DNA origami structures with a poly(ethylene glycol)-*block*-poly(L-lysine) copolymer, which is a straightforward, cost-effective, and robust route to protect DNA-based structures from degradation [72]. The self-assembly of supramolecular complexes of DNA and polymers is of relevance to gene carrier design. Trubetskoy demonstrated that template polymerization facilitates the condensation of DNA into nanoparticles. The DNA within the nanoparticles remains biologically active and can express foreign proteins inside cells [73]. Efficient gene delivery to target cells remains a significant challenge of lacking protection towards the successful development, while the excellent promise of encapsulation is a tool to protect therapeutic nucleic acids [74]. The success of gene therapy relies on vectors that can protect the nucleic acid and mediate its controlled release allowing gene expression. Peptides bear unique properties that are indispensable for gene carriers. Mann employed functional peptides to maintain the balance between DNA condensation and release. These multifunctional peptides showed high transfection efficiency with less toxicity [75]. Double-stranded RNA (dsRNA) is an important therapeutic and exhibits severe degradation without protection. Whitfield developed an efficient binding, protection, and self-release of dsRNA through the usage of a cationic polymer. The architectures of the polymer can significantly affect the lifetime of dsRNA [76].

(b) Protecting against endo/lysosomal digestion

It is commonly considered that the cationic polymers buffer the protons inside the lysosomes, which initiates endosomal escape process to avoid digestion. The ability of polyplexes (the complexations of DNA/RNA with the cationic polymers) of endo-lysosomal escape relies on the buffering capacity of polycations, and they are always evaluated by pH titer before gene transfection. Polycations are partly protonated under neutral pH, but their protonation increases within acidic endosomes, which triggers the influx of protons, as well as of chloride ions, resulting in increased osmotic pressure inside lysosomes. Additionally, the cationization of gene carriers and osmotic vesicle swelling can mediate cation-stimulated endosomal membrane disruption. Finally, the nucleic acids will be released into the cytosol. The Feliu group applied PEI as model to measure lysosomal proton buffering in situ by fluorescent pH sensor microcapsules [77]. Poly(ethylene glycol)-*block*-poly(L-lysine) highly compacts DNA into nanoparticles, showing considerable promise in human gene therapy. The Hanes group formulated pH-responsive DNA nanoparticles that mediate gene transfer via a nucleolin-independent pathway with poly(L-histidine) inserted between poly(ethylene glycol) and poly(L-lysine) to form a triblock copolymer. The inclusion of poly(L-histidine) increased the buffering capacity of polymer to levels comparable with branched PEI. Correspondently, the in vitro transfect efficiency was improved by 20-fold over poly(ethylene glycol)-*block*-poly(L-lysine) DNA nanoparticles [78]. Chitosan is one of biocompatible polysaccharides used as a gene carrier. However, the transfection efficiency of chitosan is low because of the DNA degradation in endosomes. The buffering capacity of histidine in the endosomal pH range would assist the escape of DNA from endosomes. Chang incorporated histidine into chitosan to improve the transfection efficiency. A broader buffering range of histidine-chitosan conjugation was observed, and the cellular uptake of histidine–chitosan conjugation/DNA complexes was much higher than that of chitosan/DNA complexes [79]. Charge density of BCPs contribute to good DNA condensation and high transfect efficiency while also result in heavy cytotoxicity. Poly(L-histidine) as one kind of BCP was introduced into a polyester-based gene carrier for co-delivering siRNA and doxorubicin. The polyplex showed excellent an encapsulation of doxorubicin and siRNA, as well as an initiated payload release in response to the tumor environment. The polyplex with effective endo-lysosomal escape was a verified rational approach for the co-delivery of siRNAs and chemotherapy agents for multidrug resistance reversal [80].

(c) Protecting against immune clearance

The carriers can minimize the immune response to avoid the clearance of nucleic acids by host immune system. siRNA are potent activators of the mammalian innate immune system and can induce high levels of inflammatory cytokines and type I interferons [81]. To move forward the application of siRNA in HCC, numerous methods have been developed to synthesize biocompatible polymers to minimize the immune response, including the incorporation of surface shielding segments and additional transport domains for effective and specific delivery, as well as polymers with uniform sizes and special topology [82]. Even the viral carriers, which can escape from the host surveillance, still need to be protected to avoid the innate immune response. Especially, the neutralization of pre-existing antibodies, the coxsackie, and receptor-precluded target selectivity can lead to inefficient delivery. In response to this concern, Fisher used the biocompatible polymer to protect the virus. The polymer-coated virus produces ligand-mediated uptake into cells bearing appropriate receptors, validating that adenovirus shielded with polymers is an effective method of changing its tropism and interaction with the immune system [83], which employed covalent coating and retargeting approach using a multivalent poly[N-(2-hydroxypropyl)metha-crylamide]-based hydrophilic polymer. 

### 3.2. Tumor Targeting

Off-target is problematic, because high biological activities and undesirable biodistribution of nucleic acid resulted in the loss of potency and undesired side effects. Thus, gene targeted delivery to specific organs/cells is substantial for not only efficacy but also safety. Four most common approaches will be discussed in this section, including the enhanced permeability and retention effect (EPR effect), ligand–receptor-based targeting, tissue or cell-specific release, and targeted delivery based on physical interactions.

EPR effect based passive targeted gene delivery is an important approach for effective gene therapy. The property of EPR is challenged for promoting gene delivery by nanoparticles at sites of rapid cancer growth. The common explanation of EPR concentrates on tumor blood vessel leakiness as a result of structural and architectural malformations, while the reality is that the basis of cancerous and healthy tissues with variational vascular cut-off pore sizes is incompletely understood [84,85]. Even so, EPR effect is widely exploited for tumor targeting of polyplexes if the nanocomposites have the potential of long-circulation time in blood. Targeting delivery with modest EPR effect is limited in most tumors. Sano used photoimmunotherapy (PIT) to enhance the trafficking of nanoparticles, which there was a surprisingly high leakage of nanoparticles into the tumor bed [86].

In practice, the passive targeting does not meet the requirements for gene delivery, and the demand of active targeting is rising. Since there are various types of receptors overexpressed on the surfaces of tumors, ligand–receptor-based targeting has been extensively investigated for gene delivery. In this domain, the modification of polymers is particularly attractive. Normally, the surface of polycations always displays abundant amines, which is convenient for ligands coupling. Staquicini screened a peptide library in cancer patients to reveal ligand–receptors common or specific to special vascular beds. Four native ligand–receptors were found by high-throughput analysis of a similarity search, affinity chromatography, and protein arrays [87]. Antibodies are a big category of ligand explored for cancer targeting. The basic principle that underlies antibody-targeted therapeutics is that the delivery of antineoplastic nanoparticles to cancer cells or cancer-associated tissues, such as tumor vasculature, which can be selectively increased by associating the nanoparticles that bind to receptors either uniquely expressed or overexpressed on the target cells comparing to normal tissues. With fast developments of antibody engineering, several antibodies as targeting moieties to increase the selective discharging have been administrated in clinic [88]. A rapidly growing class of biocompatible polymers use a targeting moiety to deliver potent nucleic acids selectively to malignant cells. A wide variety of targeting moieties have been used. Antibodies, aptamers, and low molecular weight organic ligands are all attracting attention. Antibody–polymer conjugates (ADCs) have the greatest success to date [89]. The Lai group studied the effects of incorporation of active targeting moieties (folate) into nanocarriers and enhanced targeting for cancer therapy [90]. The favorite method to maximize safety and efficacy is to transport therapeutics with a targeting ligand that exhibits minimized affinity for healthy tissues but high affinity for pathologic tissues. The probability of regulatory approval can conceivably be further promoted by exploiting the same targeting ligand, coupled to a biocompatible carrier, to select tumors that display sufficient targeted receptors for therapeutic efficacy [91]. Numerous synthetic methods have been developed to reliably modify cancer specific ligand moieties to polysaccharides and utilize sugars as a multifunctional building block to develop tumor targeted carriers. The design of sugar-based carrier systems has tremendous implications to preferentially target various tumor tissues through receptor interactions [92].

Tissue-specific response release is another indispensable strategy to achieve tumor targeting. Since the acidic environment of malignant tumors, the Zhu group developed a pH stimulus–responsive drug delivery carrier for synergetic cancer therapy, which is built on a triplex-DNA nano-switch capable of precisely responding to pH variations in the tumor microenvironment [93]. Beside the acidic environment, cancer cells are commonly characterized with a state of redox imbalance that compensate for oxidative stress induced by the tumor redox environment [94]. The Chang lab presented a redox-sensitive polymer/metal nanocomplex system (PSPIO) for efficient cancer theranostics. PSPIO exhibited strong redox–responsive DNA release. Due to the redox-sensitive release, the in vitro transfection efficiency of PSPIO was significantly enhanced under an external magnetic field [95]. Responsive gene release in tumor mitochondria is a prerequisite for mitochondria-targeted delivery systems to promote the efficacy of therapeutic modality. Tan and co-authors developed a mitochondrial-targeted carrier to kill tumor cells. The results showed that the nanoparticles induce mitochondria-specific heat shock to facilitate the fast variation of ROS at the same locus to eliminate cancer cells in a more effective way [96].

Polyplex-targeted delivery based on physical interactions is also very tempting if the assisted facilities are available. The typical examples of physical interactions are ultrasound and magnetic targeting. Ultrasound exposure in the presence of microbubbles increases gene transfection efficiency by numerous orders of magnitude both in vitro and in vivo. Acoustic cavitation facilitates the formation of short-lived pores in the plasma membrane. Loading microbubbles with nanoparticles capsulating nucleic acids may further improve the efficiency and specificity such that clinical trials become a realistic prospect [97]. Nontargeted and insufficient gene transfer has impeded HCC therapy. Wu investigated HCC gene-targeted delivery using the genes of suicide system and the tissue inhibitor with ultrasound-targeted microbubble destruction (UTMD). Targeted gene delivery synergistically improved the antitumor effects and may provide an effective perspective for HCC prevention [98]. Compared to ultrasound, magnetic targeting is another tool for tumor targeting. Over the past decades, the synthesis of superparamagnetic nanoparticles has been researched intensively since the nature of magnetic targeting. However, their usage in vivo is limited by their agglomeration in biological fluids. The addition of a biocompatible polymers to the surface of IONPs can stabilize these nanoparticles and attach therapeutic genes [99]. Mahajan designed superparamagnetic iron oxide nanoparticles (SPIONs) coupled with siRNA for tumor target therapy. The data showed that the nanoparticles significantly accumulated in tumors, marked by a decrease in tumor cell proliferation and an increase in apoptosis [100].

### 3.3. Enhancing the Transfect Efficiency and Minimizing the Cytotoxicity

Efficiency and safety are most important parameters for the nonviral gene carriers, which always guide the development of gene therapy. Generally, proton buffering capacity [101], high charge density [102], high molecular weight [103], amphiphile [104], cell penetration molecules [105,106], and tumor targeting [107] contribute to the high transfect efficiency. On the contrary, high charge density, high molecular weight, and amphiphile usually result in unwanted cytotoxicity. The biocompatible polymers play a central role on enhancing the transfect efficiency while minimizing the cytotoxicity. 

Endo-lysosomal escape of gene carriers is crucial to enhancing the efficacy of their payload, and the proton buffering capacity is a powerful tool to modulate the transfect efficiency [108,109,110]. Since we already discussed these above and skip them herein, high charge density and high molecular weight normally contribute to the condensation of nucleic acids and result in good transfect efficiency, while excessive charge density makes them toxic for biological applications. The Saltzman lab synthesized a series of terpolymers of low charge density with high molecular weight. The gene delivery of screened terpolymer showed highly efficient to tumor xenografts, and the data displayed that the tumors were inhibited significantly with minimal cytotoxicity in vitro, as well as in vivo [49]. Cytotoxicity of the cationic terpolymer was minimized with reduced charge density while the efficiency was promoted via increasing molecular weight and hydrophobicity [101], which indicating the molecular weight and amphiphile nature of polymer facilitate the gene trafficking in cells. In the past decades, various methods have been employed to prepare amphiphilic polymers to enhance gene delivery [111,112]. The Grinstaff lab developed a new approach to prepare a gene carrier, which can transform from a cationic to an anionic amphiphile intracellularly. Enhanced gene transfection of a charge-reversal amphiphile was observed compared to conventional cationic amphiphiles [113]. 

Before accumulated to the tumor, polyplexes will confront all kinds of biological barriers during the journey. Cellular uptake is one of barriers for polyplexes, and the ability of cell penetration is very crucial for gene carriers. The Liu lab discovered surface mutagenesis of proteins in a manner that significantly increases their net charge, which can penetrate a variety of mammalian cell lines resulted in efficiently gene silence [114]. Beside the natural cell penetrating proteins, synthetic materials also widely developed. The Wang lab prepared cell penetrating peptide-based polyplexes decorated with polysaccharide to improve gene transfection. Due to the effective cellular uptake efficiency, the transfection efficiency was much higher than the correspondent polyplexes without the ability of cell penetration [115], and the incorporation of penetrating molecules into biocompatible polymers as the skeleton of the carrier is still popular. Khan system reviewed the recent updates of cell-penetrating peptide-based materials for gene delivery [116]. Although tumor targeting is very important parameter for biocompatible polymers to boost the efficiency of gene delivery without sacrifice of the cytotoxicity, we have already discussed this above and will skip it here.

Viral vectors possess the satisfied efficiency while they have safety concerns. Virus mimicking has become a promising direction for the design of nonviral carrier. To achieve biomimicry of a virus, virus-like nanoparticles were generated to deliver various nucleic acids to the cytoplasm of cells specifically in vivo [117]. Synthetic gene vectors usually have a net positive surface charge, which enables the condensation of nucleic acids, adsorption-mediated cell binding, and internalization. Generally, the transfection efficiency of the current generation of synthetic materials is poor. Due to the mechanism of very efficient cell entry and immune escape, the transfection efficiencies of the viral vectors remain unprecedented. The virus-mimicking of synthetic materials has become a very hot in the domain of nonviral gene carriers [118]. Aoyama and co-authors fabricated a saccharide nanoparticle derived from a macrocyclic glycocluster compound. As a novel of artificial glycol carrier, it compactly packed DNA into virus-like nanoparticles. The polyplexes are well charge-shielded and efficiently transfect in vitro [119]. The TAT peptide in the HIV-TAT protein is responsible for the translocation of the HIV nanoparticles and has been conjugated in a variety of artificial polymers to transport them to across the cellular membrane. However, the cationic nature of the peptide does not allow for exhibiting these peptides on the surface of the polyplex. The Thayumanavan lab developed a novel molecular design to guarantee a TAT peptide on the surface of the polyplex. Consequently, the gene expression was significantly enhanced [120].

The cytotoxicity of polymers is dosage-dependent, and the usage of polymers for gene carriers is always an extremely low concentration. Although the polymers will be accumulated in the body, the biocompatible polymers are often degraded fast after the therapeutics, reaching the site of the tumor. Therefore, the application of biodegradable polymers can be deemed a tool to minimize the cytotoxicity of gene carriers.

### 3.4. Minimize the Unwanted Immune-Response

Proteins, including cytokines, chemokines, antigens, antibodies, and other functional proteins, can trigger specific immune response and are traditional drugs for cancer immunotherapy. However, these endogenous proteins have high immunogenicity and are cleared quickly by host immune system. For example, antibodies often bind to the specific or nonspecific proteins in plasma, which will reduce or eliminate their effects. On the contrary, gene drugs packed by polymers or lipids are substantial stable. mRNA is typical drug, and customized mRNA can translate the correspondent protein. Compared to mRNA, therapeutic proteins have many disadvantages. First, proteins have a bigger size than the corresponding mRNA, and it is difficult to condense them into nanosizes for delivery. The unprotected proteins often trigger unwanted immune responses in vivo. Second, therapeutic proteins are not stable, and they often aggregate in blood [121]. When mRNAs are chosen as drugs, they are easier to be protected with nonviral carriers [122] to avoid the degradation by host enzymes [123]. Ulkoski and co-authors developed endosomolytic polymers for mRNA delivery. The structure–activity relationship demonstrated that the mRNA encapsulation efficiency is modulated by the cationic density and shorter alkyl side chains. The high-throughput approach they developed can accelerate the screening of polymeric systems to assess various carriers for mRNA delivery [124].

## 4. Gene Delivery by BCPs for HCC Immunotherapy

Immunotherapy has become an important intervention for HCC, and nucleic acids as a category of agents play an indispensable role in immunotherapy, such as mRNAs, siRNAs, and anti-sense RNAs. In other words, the gene modulation is an important tool for immunotherapies. 

### 4.1. mRNA Vaccine for HCC

Beside to minimize the unwanted immune response, BCPs can amplify the immune response and active the immune cells to promote the immunotherapy of HCC. Based on the development of mRNA vaccine in malignant tumors in the past decades, the SARS-CoV-2 mRNA vaccine was fast passed through the clinic trials and played a special role in helping to slow down the COVID-19endemic [125]. Therefore, mRNA vaccines are attracting more and more attention compared to conventional vaccines due to their high potency, safety, ability for rapid development, and low cost [123]. Currently, various mRNA therapeutics have reached a milestone at high speed in the immuno-oncology field. For a long time, the major interest in the use of mRNA was on the development of cancer vaccines using mRNA encoding tumor antigens to active lymphocytes in vivo. Due to the smart design of both the structures of mRNAs as well as gene carriers that improve their in vivo stability and targeting, the therapeutic potential of mRNA in cancers can be considered as endless. Eventually, a tremendous amount of novel immunotherapeutic approaches concentrates on the use of mRNA beyond their use as the source of tumor antigens [126]. Synthetic custom mRNA provides a template for protein with interested sequences, and proteins lay the footstone for a broad range of pharmaceutical applications, including various modalities of cancer immunotherapy. Nucleoside modification and elimination of double-stranded RNA can avoid the immunomodulatory activity of mRNA and increase/prolong the productions of protein therapeutics. With the help of nanoparticle-based formulations that increase transfection efficiency and facilitate lymphocytes or tumor targeting, nucleoside-modified mRNA enables efficient transport of cytokines, chemokines, costimulatory receptors, antigens, or therapeutic antibodies [127]. The identification of suitable specific antigens to the tumor for cancer vaccines is still a challenge. Alternative processing of mRNA may offer the potential of a broadened target space and analysis of mRNA processing events in cancer cells with an emphasis on mRNA splicing have been extensive investigated. Of course, many bottlenecks must be overcome for this new avenue to have clinical translation [128]. Matsui and co-authors confirmed that Heat Shock Protein 70 (HSP70) was highly expressed in HCC by immunohistochemical staining. They have delivered a HSP70 mRNA to dendritic cell (DC) for treating unresectable or recurrent HCC. The phase I and II trials have verified the safety and efficacy of this DC therapy. Especially, the OS of the DC group was significantly longer than the control groups [129]. 

### 4.2. Adaptive Immunotherapy for HCC

Adaptive immunity-based therapy, including checkpoint blockade inhibition, CAR-T, TCR-T, and B cells, are widely developed for the treatment of cancer. TCR-T is extensively developed for cancer therapy, while TCR engages with both tumor intracellular and surface antigenic peptides embedded in the major histocompatibility complex (MHC) comparing to CAR [130]. B cells are associated with survival and immunotherapy response, and B-cell-based therapy has been developed recently, while the generation of good practice manufactured B cells is still facing various obstacles [131,132]. Therefore, there is still a long way to go for both TCR-T and B-cell therapies to clinic applications. More importantly, they are seldom exploited for liver cancer, and biocompatible polymers are not involved much in these therapies currently. Herein, we mainly discuss the CAT-T and checkpoint blockade therapies in the domain of adaptive immunotherapy. Table 2 has listed the current immune cells applied (or potential) for HCC immunotherapy.

#### 4.2.1. Check-Point Blockade Based Immunotherapies

**Figure 3:**
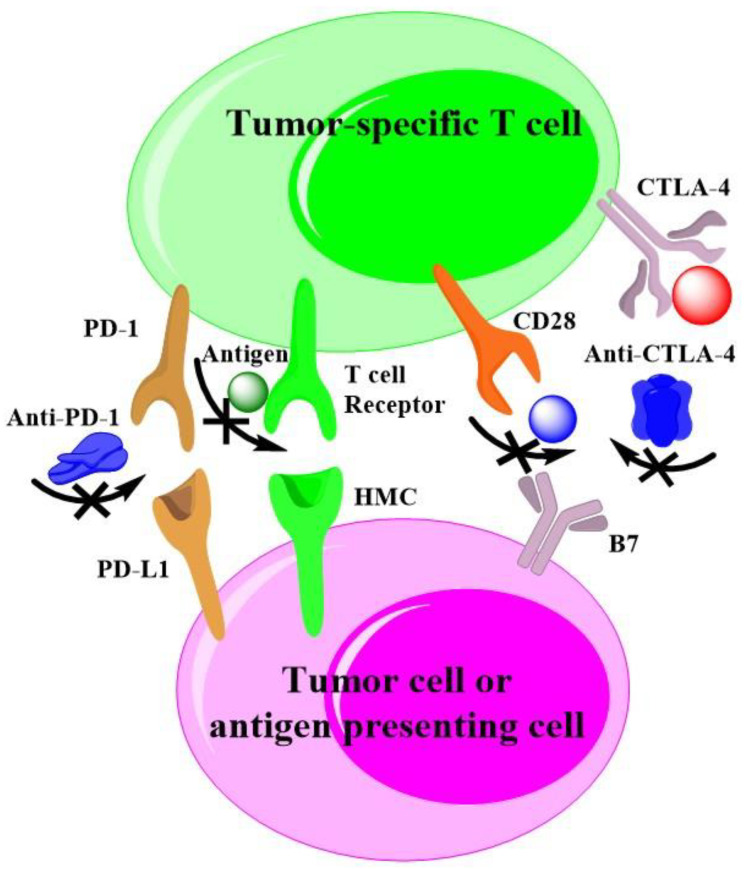
Checkpoint-based cancer immunotherapies.

Agents to inhibit the immune checkpoint receptors or their ligands have revolutionized the treatment of diverse malignant tumors. Many tumors are recognized by adaptive immunity, but these adaptive responses can be blocked by immunosuppressive mechanisms within the tumor. A few novel approaches are striving to expand actions of immunotherapy, which include targeting alterative immune checkpoints [133]. Currently, the checkpoints of programmed cell death protein 1(PD-1)/programmed cell death ligand 1(PDL1) and cytotoxic T lymphocyte antigen-4 (CTLA4) are widely exploited for cancer immunotherapy. Drake and co-authors have systematic review the cancer immunotherapy as melanoma, lung and kidney cancer [170], they have presented the mechanism of action as to check-point inhibition by specific antibody. As shown in Figure 3, we also plotted out the common mechanisms of action of checkpoint-based cancer immunotherapies with help of the reference [170], that also applied to HCC.

As early as 1987, Brunet and co-authors validated a protein belonging to the immunoglobulin superfamily, named CTLA-4. It is mainly expressed in activated lymphocytes and contributes to T-cell-mediated cytotoxicity in inducible models of the process to taking part in cell–cell recognition [134]. With the recognition of immunotherapy, CTLA-4 has been widely investigated in preclinic and clinic. Alegre and co-authors believe CTLA-4 ligation raised the threshold amount for T-cell activation and arrested T-cell cycle progression [135]. Zappasodi and co-author researched the effect of CTLA-4 blockade on the metabolic fitness of intratumor T cells in relation to the glycolytic capacity of cancer cells, finding that CTLA-4 blockade promotes metabolic fitness and the infiltration of immune cells. Notably, the responses of tumor-specific CD8^+^ T cell are correlated with the phenotypic and functional destabilization of tumor-infiltrating regulatory T cells [136]. Yang and co-authors reported CTLA-4 expression in B-1a cells as a substantial function in maintaining self-tolerance by modulating these early-developing B cells that express an enriched repertoire for autoreactivity, showing that the CTLA-4 regulation of B-1a cells is a key immune regulatory mechanism [137]. Recently, CTLA-4 has become a major targeting site for cancer therapy. Consequently, monoclonal antibodies (mAbs) and CTLA-4-siRNA were developed to inhibit the expression. Esmaily and co-authors silenced CTLA-4 in tumor-infiltrating T cells by siRNA-loaded chitosan–lactate, which resulted in tumor regression and increased mice survival. Compared to the treatment of tumor-bearing mice with DC vaccine, the combination of siRNA-loaded NPs and DC vaccine exhibited synergistic antitumor effects [138]. However, the clinical trial with CTLA-4 inhibitors alone for advanced HCC are disappointed. For example, the administration of tremelimumab in patients with HCC revealed a partial response rate of 17.6% and disease inhibition rate of 76.4% [139]. Probably, biocompatible polymers will act as indispensable roles to enhance the immunotherapy with CTLA-4 siRNA.

The PD-1/PDL1 axis is another targeting site for cancers, as well as HCC immunotherapy. PD-1 plays a crucial role in inhibiting immune responses and promoting self-tolerance through regulating the activity of T cells, mediating the apoptosis of antigen-specific T cells and blocking the apoptosis of regulatory T cells. PD-L1 is a trans-membrane protein that is recognized to be a co-inhibitory factor of the immune response. It can bind to PD-1, resulting in reducing the proliferation of PD-1 positive cells, inhibiting their cytokine secretion, and inducing apoptosis. The PD-1/PD-L1 axis is responsible for malignant tumor immune escape and makes a significant effect on cancer therapy [140]. To block the PD-1/PD-L1 axis, mAbs are exploited, and some of products have been applied in clinics [141]. The clinical efficacy of PD-1 suppression and its ability to augment the effector function of the tumor-specific CD8^+^ T cells PD-1/PD-L1 inhibition ratio have broadened the opportunities for therapy in patients with previously untreatable malignancies or ineligible to traditional therapies [142]. However, a clinical response to anti-PD-1 antibody is rare (<5%) for the treatment of HCC [143]. New methods are much urgently needed to promote the efficiency of anti-PD-1/PD-L1 axis therapy, and siRNAs to silence PD-1 or PD-L1 have great promise. Since PD-L1 overexpresses on the surface of tumors while PD-1 is an inhibitory receptor that is expressed by all T cells during activation [144], the targeting delivery system often employs the PD-L1 siRNA to break the PD-1/PD-L1 axis [145]. Zhu and co-authors developed a nanomaterial encapsulating doxorubicin and PD-L1 siRNA to evaluate its antitumor effects on HCC. The results shown that PD-L1 siRNA significantly inhibited the tumor volume through silenced the expression of PD-L1 in tumor tissue of a H22 tumor-bearing animal model. Additionally, the treatment of PD-L1 siRNA also modulated the populations of matured dendritic cells and cytotoxic T cells in tumor tissues [146].

#### 4.2.2. CAR-T Cell Therapy for HCC

CAR-T cell therapy in early clinical trials revolutionized cancer therapy, especially the patients with pre-B-cell acute lymphoblastic leukemia or B-cell lymphomas. These trials resulted in rapid FDA approvals of anti-CD19 CAR T-cell products for both acute lymphoblastic leukemia and coupled types of B-cell lymphoma [147], although CAR-T cell therapy has achieved successful outcomes against hematological malignancies and provided a new perspective for treating solid tumors. However, the low efficacy of CAR-T cells for solid tumors stops its further clinic applications, and it is very urgent to update CAR-T cell therapy for solid tumors [148,149]. To our best knowledge, there are only two mostly positive trials reports that have used GD2 CARs to target neuroblastoma [150] and HER2 CARs for sarcoma [151]. The reason is not yet clear, and there is a lot of controversy. The solid tumor landscape presents unique barriers comparing to hematological malignancies. The CAR T cells must successfully traffic to solid tumor sites and successfully infiltrate the stromal elements of solid tumors in order to induce tumor-associated antigen (TAA)-specific cytotoxicity, regardless of antigen loss or heterogeneity. Additionally, T cells must surmount challenges from the microenvironment of solid tumors, such as nutritional depletion, hypoxia, the presence of suppressive cytokines, and suppressive immune cells [171].

**Figure 4:**
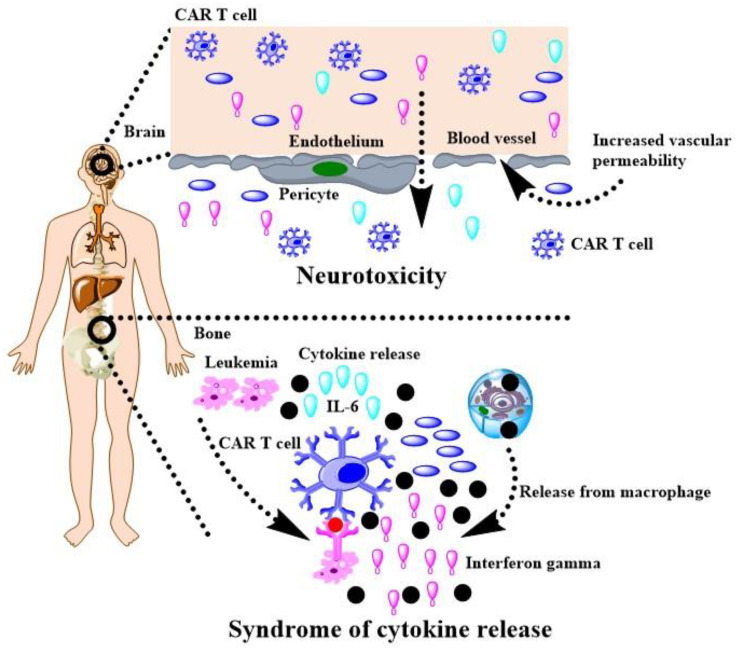
CAR T cell therapy for cancers and its challenges.

Nanotechnologies with biocompatible polymers are potential solutions to crack down above matters. Parayath and co-authors delivered CAR mRNA into circulating T cells for transiently reprograming to recognize disease-relevant antigens. In mouse models of prostate cancer and hepatitis B-induced HCC, repeated infusions of these nanomedicine induce sufficient host T cells expressing tumor-specific CARs to cause tumor regression at levels similar to bolus infusions of ex vivo engineered lymphocytes [172]. Moffett and co-authors have developed PGA based polymers to deliver mRNA for cancer treatment and demonstrated CAR-programmed T-cells with appropriately designed mRNA nanoparticles can transiently program gene expression to improve their therapeutic potential [173]. Actually, smart biodegradable polymers have the potential to overcome the matter confronted the CAR-T therapy in solid tumor. A tumor microenvironment imposes barriers to the passive diffusion of CAR-T mRNA, which renders tumor penetration an unresolved obstacle to an effective active of T cells, while the tumor penetrated polymeric nanocomposites can enhance the trafficking of drugs [174], as well as applied for CAR-T mRNA. Hypoxia plays a crucial role in cancer progression, immune editing, and drug response, which often results in tumors escaping from immunosurveillance and CAR-T cell-mediated cytotoxicity [175]. Nguyen and co-authors have demonstrated that oxygen delivery through polymeric microcapsules is dependent on multiple parameters, such as polymeric shell, the shell thickness, the pressure gradient across the shell, and oil layer between the polymeric shell and the gas core [176]. These polymeric microcapsules have chance to promote the efficacy of the CAR-T mRNA for solid tumor. Of course, CAR T cell therapy has many challenges, such as cytokine release syndrome and neurotoxicity during treating leukemia and lymphoma [177]. As shown in Figure 4, we have drawn a scheme with help of the reference to interpret the side effect [177], which should be addressed when gene intervention-based CAR T cell therapy is developed in HCC.

### 4.3. Innate Immunotherapy for HCC

Beside the adoptive immunotherapy, innate immunotherapy also has bright future for HCC treatment, especially the therapies based on natural killer (NK) cells, macrophages, and neutrophils.

So far, T cells-based cancer immunotherapies, including immunological checkpoint blockade and adoptive cellular therapy, have attracted the main attentions of immunotherapies. However, NK cells are receiving renewed interest recently since they present the considerable advantages of not relying on antigen specificity [178]. Several groups have successfully developed NK cell functions directed against glioblastoma [152], neuroblastoma [153], lung cancer [154]. Compelling evidence suggests that NK cells play an irreplaceable role in the immune function of the liver and immunotherapy against HCC, indicating that NK cells might be an ideal target to prevent HCC [155]. NK cells are essential components of innate immunity against tumor and vary in phenotype, and the functions have been described in HCC patients, who show disruption of NK activating receptor/ligand axis. The CAR-engineered NK cells provide unique opportunities to create CAR-NK with multiple specificities with potentially less adverse effects [156]. Nath and co-authors have verified that NK Cell recruitment and activation are regulated by CD47 expression in the tumor microenvironment [157], which make it possible to treat cancers with CD47 mRNA targeting delivery to NK cells. Au and co-authors have developed tri-specific natural killer cell nano-engagers for targeted chemoimmunotherapy, which employed biodegradable poly(ethylene glycol)-*block*-poly(lactide-*co*-glycolide) (PEG-PLGA) to co-deliver anti-human EGFR antibody, anti-CD16, and anti-4-1BB to treat B16F10 tumor-bearing mice [158]. It has demonstrated that anti-cancer activities of NK-92 cell line are excellent in clinical trials. While the clinical efficacy of NK-92 cells has not reached their full potential because of reduced immune functions compared to primary NK cells. Enhancements of NK-92 functions currently rely on gene delivery (including mRNA and plasmid DNA) with limited efficiencies. To enable precise genetic modifications, CRISPR genome engineering platform for NK-92 based on the nucleofection of CAS9 ribonucleoprotein was developed [159]. Furthermore, polymer-stabilized CAS9 nanoparticles and modified repair templates increase genome editing efficiency to active the functions of NK cells [160]. Based on the crucial role of NK cell in the therapy of HCC, BCPs for gene delivery (including plasmid DNA, mRNA, miRNA, siRNA, and CRISPR/CAS9 mRNA) to NK cells to fight HCC show bright future. 

Macrophages have commonly been categorized into M1 or M2 polarized phenotypes. Pro-inflammation M1 classically activated by IFN-γ or lipopolysaccharide [161]. The M1-polarized macrophages secrete IL-6, TNF-α, and other tumor-inhibition cytokines. Immunosuppressive M2 alternatively activated by interleukin IL-13 or IL-4. The M2-polarized macrophages secrete alternative macrophage activation-associated chemokines and promoting angiogenesis. Tumor-associated macrophages (TAMs) promote carcinogenesis by stimulating angiogenesis, migration, invasion, and metastasis [162]. TAMs are abundant in the tumor microenvironment of HCC, and better understanding of tumor associated macrophages would allow for the development of novel macrophage-targeting immunotherapies [163]. Although it is still controversy the role of M1 in the development of HCC, most target is to polarize the macrophage from M2 to M1 in tumor environment. For example, IL-37 was drugged to suppresses HCC growth through inhibiting M2 polarization of tumor-associated macrophages [164]. At this juncture, gene delivery to polarize M2 to M1 in tumor environment would be especially important. Poly(glutamic acid) was used to targeted deliver mRNA to TAMs and then reprogrammed them toward an M1 phenotype, which could thwart their pro-cancer activities and unleash antitumor immunity [165]. Sharma and co-authors have employed single cell RNA sequencing to extensively analyze the cellular landscape of human liver from development to HCC, which provide novel targets for interventions in HCC [166]. Based on the extensive investigations of TAM biology, various gene therapeutics (including mRNA, siRNA, and miRNA) are ready for reprograming M2 to M1 in the HCC tumor environment if smart and safety polymeric carriers are available. 

Neutrophils are the most abundant white blood cells in blood, as well as constitute a significant part of the tumor microenvironment. Neutrophils play major roles associated with inflammation and are actively involved in cancer progression and metastasis [167,179]. The ratio of circulating neutrophil-to-lymphocyte as a robust biomarker represents clinical outcome in various cancers. The phenotypes of tumor-associated neutrophil (TAN) can predict cancer development and progression. Various treatments on TANs obviously affect therapeutic efficacy [168]. Neutrophils have a significant impact on the tumor microenvironment through cytokines and chemokines secreted by TANs, which influence inflammatory cell recruitment and activation. Moreover, products generated by neutrophils, such as proteinases and reactive oxygen species, have specific roles in regulating cancer cell proliferation, angiogenesis, and metastasis. Therefore, TANs targeting as a tool of antitumor therapy is reliable [180]. Although miR-223 was targeted into neutrophils to enhance the clearance of infectious diseases [169] and nanoparticle targeting of adherent neutrophils to prevent vascular inflammation with more and more attention to the neutrophils exploiting in HCC therapy [181]. Therefore, the demands of BCPs for gene delivery to neutrophils to fight HCC are about to be expanded.

### 4.4. Intervention of Oncogenes to Modulate Tumor Immune Microenvironment 

**Figure 5:**
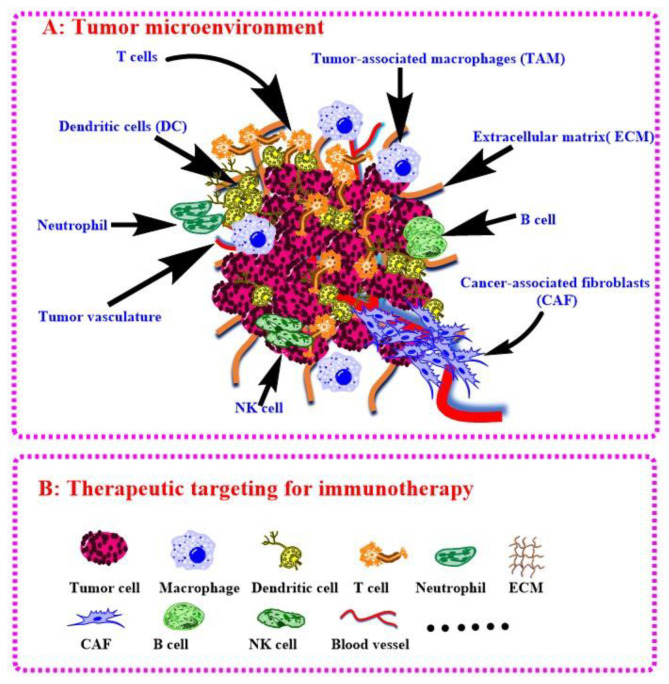
Tumor microenvironment (**A**) and therapeutic targeting for immunotherapy (**B**).

TIME often decides the tumor progressive and their response to immunotherapy [141]. Anti-PD1/PDL1 therapy shows bright future in the treatment of HCC while it is only response <15% patients because of the harsh tumor environment. Fortunately, intervention of oncogenes can steadily modulate TIME. Zhao and co-authors have validated PTEN mutations resulted in immunosuppressive in glioblastoma based on genomic and transcriptomic analysis [182], Triulzi and co-authors investigated the correction between HER2 activity and TIME, and the results shown that activated HER2 oncogene modulates recruitment and activation of tumor infiltrating immune cells [183]. Actually, the above oncogenes also involved in HCC and can be developed for HCC immunotherapy [184,185]. Of course, aiming at the host cell of target oncogene is the next step for the modulation of TIME to boost HCC immunotherapy. Joyce group has systematic reviewed the therapeutic targeting of the tumor microenvironment recently [186]. They have summarized the most advanced tumor microenvironment associated therapies, discussed the current challenges, and presented future perspectives in this evolving field. As shown in Figure 5, we have plotted out the immune cells in tumor environment referring their work, which can be exploited as target for HCC immunotherapy. In this article, we have reminded immunotherapies of some other tumors via gene delivery with BCPs because they are good references for HCC. For example, the modulations of KRAS in lung cancer and MYC in HCC are useful for TIME-positive changes for immunotherapy, while the process of target delivery by BCPs is not big different, which are just variational organs and corresponding physiological characteristics. However, the malignant tumors still have more common properties, and BCPs can be modified with respective ligands to achieve targeting (e.g., glycyrrhizic acid for HCC while folic acid for lung cancer). Therefore, the success applications of BCPs in other tumors can accelerate the research of HCC immunotherapy with nucleic acid delivery by BCPs.

The MYC gene is widely investigated in HCC, and its high expression can worsen the TIME, which is not conducive to anti-PD1 therapy. We believe that MYC gene inhibitor (MYCi)-based drugs can promote the responsiveness of anti-PD1/PDL1 treatment mainly for the following reasons. First, MYC gene directly or indirectly regulates about 15% of human genes [187,188], many of which are on the joints of immune-related signaling pathway. Second, MYC gene can regulate immune-related signaling pathways and ameliorate TIME. Casey and co-authors found that the MYC gene regulates the expression of CD47, PD-L1, and genes associated with immune signaling pathways, which ultimately makes cancer patients resistant to PD1 treatment [189]. Third, anti-PD1/PDL1 therapy was found to be promoted after the use of MYCi to block MYC gene expression. Han and co-authors screened a MYCi and found MYCi increased the responsiveness of anti-PD1 therapy by inhibiting MYC gene expression [190]. There are a lot of investigations on the activation and inhibition of MYC gene, which makes it easy for us to choose gene drugs. In this case, it is feasible to use MYCi and PDL1-siRNA as drug to target tumors for combination therapy. MYC gene is closely related to tumor development and treatment, and it is regarded as the most promising drug target to promote HCC immunotherapy because MYC is disordered protein and lack of available drug identification site. Looking for drugs acting on MYC protein has been a major problem in drug research and development, and MYC gene regulation has been widely studied in order to develop reliable gene drugs. Ma and co-authors showed that lncRNA HOTAIR activates MYC gene expression through negative regulation miRNA-130a [191]. Shigeyasu and Cho found that PVT1 lncRNA activates MYC gene expression [192,193]. Yu and co-author found that circBIRC6 can positively regulate MYC gene expression [194]. In the case of MYC gene inhibition, Tai and co-authors found that miR-342-3p inhibits MYC gene activity by inhibiting the expression of E2F1[195]. Weissmiller and co-authors found that the SMARCB1 gene directly inhibits MYC gene expression [196]. The above investigations have verified that TIME regulation by MYCi can promote anti-PD1/PDL1 treatment. At this juncture, the delivery system become especially important and biocompatible polymeric gene carriers will play an irreplaceable role for immunotherapy of HCC. 

### 4.5. Intervention of Metabolism to Modulate Tumor Immune Microenvironment

**Figure 6:**
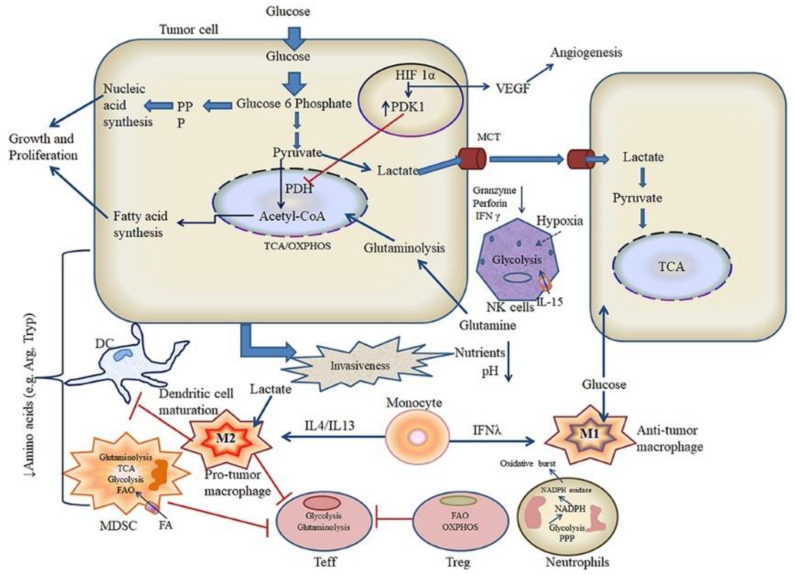
Metabolic crosstalk of tumor and immune cells in the tumor microenvironment ([207] *Front. Oral Health* **2020**, *1*, 585710). Copyright © 2020 Chaudhary, Bag, Arora, Radhakrishnan, Mishra and Mukherjee.

The microenvironment in cancerous tissues is immunosuppressive, whereas the microenvironment of tissues affected prognosis of immunotherapy. Although these opposing immunological states, the metabolic states in the tumor microenvironments and inflammatory diseases are similar, which show elevated levels of metabolic by-products while have low levels of nutrients compared with normal tissues. A clear understanding of the metabolic signature of HCC will enable therapeutic intervention aimed at reprograming the bioavailability of metabolites and modulating the dysregulated immunological state, promoting the immunotherapy [197]. As discussed above, reprograming of TAMs was widely developed for HCC therapy. Recent investigations have indicated that metabolism profiles manipulate phenotypes and functions of macrophages. On the contrary, polarization can trigger metabolic shifts in macrophages. Those discovery implicate a special role of metabolism in TAMs, and it can be target for the promotion of immunotherapy [198]. The research of immune metabolism has revealed that metabolic changes can result in anti-cancer immunity. Correspondingly, combination therapies with metabolic inhibitors and antibodies of immune checkpoint blockade have shown exciting results. The Rathmell lab developed strategies to shift immune cell metabolism to tune TIME, and finally to enhance immunotherapy [199]. Regulatory T cells (Tregs) are a subset of T cells that contribute to immunosuppressive effects in tumor microenvironment, which can promote differentiation, proliferation, secretion of immunosuppressive factors, and chemotactic recruitment of Tregs to play crucial role in immunosuppression in tumor tissues. The cell metabolism reprogramming is relative to the functional effects on Tregs. Therefore, it’s important to well understand the role of cell metabolism on the TIME for HCC immunotherapy [200]. The knowledge from extensive research in immune metabolism shows that targeting metabolism could help to enhance antitumor immunity [201]. The Locasale lab developed a computational pipeline to study metabolic programs in single cells to define the intratumor metabolic landscape. They found the expression of both glycolytic and mitochondrial network strongly correlates with hypoxia in all cell types, especially the immune cells [202]. Metabolic pathways could modulate the TIME and mitochondrial metabolism, which are an attractive target for cancer immunotherapy. Rosner lab have verified that BTB and CNC homology1 targets mitochondrial metabolism [203]. Glycolysis level correlates with immune activity in TIME, while the systematic investigation of the relevance between tumor glycolysis and tumor immunity in various tumor remains scarce. Jiang and co-authors have found glycolytic activity enhances PD-L1 expression on tumor cells, and subsequently promotes the response of anti-PD-1/PD-L1 immunotherapy [204]. Targeted delivery of therapeutics to mitochondria remains a great challenge due to their location in the sub-cellular compartment and complexity of the intracellular environment. Jiang and co-authors have reported a class of mitochondrion-targeted liposomal delivery carriers, which exhibits about 3.7-fold higher mitochondrion-targeted delivery efficacy than current triphenylphosphonium [205]. Metabolism regulation of tumor and simultaneously modulating the TIME to perform immune attack are significant for cancer prevention. Liu and co-authors have developed a novel drug vector to inhibit glycolysis of cancer cells and mitigate the immunosuppressive microenvironment [206]. Chaudhary and co-authors have reviewed recent literatures on metabolic reprogramming and associated signaling pathways that mediate crosstalk of tumor with immune cells [207]. As shown in Figure 6, they have provided a scheme as to metabolic crosstalk of tumor and immune cells in tumor microenvironment. Although they mainly discussed in oral squamous cell carcinoma, while it’s a good reference for HCC. 

## 5. Summary and Future Perspectives

HCC is an incurable disease, while immunotherapy mediated by gene intervention displays a tremendous potential. The therapeutics of nucleic acids, especially mRNA, siRNA, miRNA, and crisper/CAS9 mRNA, have been widely investigated for HCC treatment in preclinic and clinic. Indeed, once the sequences of interest nucleic acids are customized, the clinic outcomes are mainly dominated by the delivery systems. The viral carriers, including adeno-associated virus (AAV), lentivirus, and retroviral vector, still have serious concerns on the immune and insertional mutagenesis [208,209]. In the category of nonviral gene carriers, BCPs take great promise to carry all kinds of genetic theraputics to fight HCC. Biocompatible cationic polysaccharides, polypeptides, and polyesters have gained more and more attention due to their unique properties of biology and physiochemistry. Herein, we would like to address some issues as to BCPs for gene delivery. 

(a) The development of cancer biology, immunology, and molecular biology will provide increasing numbers of targets or options for genetic therapeutics. Correspondently, arising parameters will need to be considered when gene carriers are being designed. At the early stage, plasmid DNA was exploited as drug for gene therapy [210]. Around 2000, gene silence was extensively investigated, and RNAi became an option [211]. Afterward, epigenetics become a hot domain and miRNAs have been functionalized to silence or activate the expression of target genes [212]. mRNA drug is a little bit complex, which appeared in 1990 or earlier [213], while it became “hot” drug for cancer vaccine 10 years ago [123]. With the validation that gene editing is a robust tool for gene intervention, CRISPER/CAS9 have been exploited as emerging drug for diversity therapies [214]. Biocompatible polymers as nonviral gene carriers are gaining recognition for their potential in avoiding immunogenicity and mutation problems inherently associated with the use of viral vectors. A dozen years ago, the degradation nature of the polymer can be exploited as a tool to release the plasmid DNA into the cytosol, which contributes to gene therapy [215]. In the future, more and more novel genetic therapeutics will be emerged. In compliance with demand of biology, the polymer scientists should keep the synthesis updated to generate more novel materials. 

(b) Gene carriers with nature of safety, targeting, high efficiency, spatiotemporal release will continue being wanted. To address the safety concern, biocompatible polymers have been exploited since the cytotoxicity of carriers is dosage dependent and biodegradable gene carriers can avoid high cytotoxicity. To address the issues of therapeutics off-target, bioconjugate chemistry, which can contribute to the targeting of nanoparticles, were significantly developed. To address efficiency of gene carriers, the corrections between charge density/molecular weight of polymers and efficiency were extensive investigated. To address spatiotemporal release of gene carriers, the relationship between the architectures of polymers and release kinetics were researched. However, there are still many matters need to be fixed for better gene therapy for HCC.

(c) Although gene intervention mediated immunotherapy plays a crucial role in the process of HCC prevention [216], the HCC patients with higher OS in clinic got the benefits from combination therapies [217]. The chemotherapy still is the indispensable tool to prevent HCC in clinic [218]. Some chemicals (e.g., Lenvatinib) can remove the cancer-associated fibroblasts and tumor angiogenesis [219], which contribute to cancer immunotherapy with gene intervention. The antibody-resistance is one of most urgent matters to cancer immunotherapy, while there are growing cases from both preclinical studies and clinical observations, which are verified that radiotherapy could be a powerful driver to augment the efficacy of immune modulations because of its ability to activate the antitumor immune response and potentially to mute resistance [220]. Therefore, HCC immunotherapy must be combined with other treatments, such as radiotherapy and chemotherapy.

(d) The gene intervention with BCPs for immunotherapy in all kinds of tumors can be referred by HCC. Up to date, all kinds of solid tumors have conducted immunotherapy by gene intervention. Although there are many differences between HCC and other tumors, they have much more similarities. For example, anti-PD1/PDL1 therapy is developed not only for HCC, but also for a wide variety of other cancers (e.g., breast cancer, lung cancer, brain cancer, and gastric cancer). To accelerate the development of gene intervention in HCC immunotherapy, it is very important to refer the knowledge of other cancer immunotherapy with gene intervention using BCPs.

## Figures and Tables

**Table 1:** Categories of polymers and their applications for nucleic acid delivery.

Categories of Polymers	Advantages	Disadvantages	Drugs Applied	Cancers Applied	References
Polyanhydrides	Biodegradable	Fast degradation, difficult to incorporate positive charge	pDNA, et al.	N/A	[23]
PAMAM	Relatively high transfect efficiency, biodegradable	High cytotoxicity	mRNA, CRISPER/CAS9, miRNA, siRNA, pDNA, et al.	Liver cancer, brain cancer, breast cancer, gastric cancer, ovarian cancer, lung cancer, head and neck cancer, et al.	[24,25]
PEI	Relatively high transfect efficiency	High cytotoxicity, non-biodegradable	siRNA, pDNA, et al.	Liver cancer, et al.	[26,27]
PDMAEMA	Relatively high transfect efficiency	High cytotoxicity, non-biodegradable	siRNA, pDNA, et al.	Liver cancer, et al.	[27,28]
Polysaccharides	Biodegradable, good solubility	Difficult to synthesize, difficult to characterize	siRNA, pDNA, et al.	Liver cancer, et al.	[27,28,30,31,32]
Polypeptides	Biodegradable, good solubility, higher architectures	Difficult to synthesize, low transfect efficiency, high immunogenicity	CRISPER/CAS9, siRNA, pDNA	Liver cancer, lung carcinoma, et al.	[33,34,35,36,37,38]
Polyesters	Biodegradable	Fast degradation, difficult to incorporate positive charge	mRNA, siRNA, pDNA, et al.	Liver cancer, lung cancer, brain cancer, et al.	[39,40,41,42,43,44,45,46,47,48,49,50,51]

**Table 2:** Current immune cells applied (or potential) for HCC immunotherapy.

Categories of Immune Cells		Statues	Challenges Encountered	If Biocompatible Polymer Applied for Cancer Immunotherapy	Applied Cancers (Including Clinic and Preclinic)	References
Adaptive immunity	TCR-T	Is developing for solid tumors.	Substantial toxicity	Yes	Melanoma, et al.	[130]
	B cell	Is developing for solid tumors and hematological malignancies.	Difficult to generate manufactured B cells	Not yet	Lymphoma, melanoma, breast cancer, et cal.	[131,132]
	Checkpoint blockade	Applied in clinic for various cancers, continue to be developed	Drug resistance, only sensitive to about 15% patient	Yes	Liver cancer, et al.	[133,134,135,136,137,138,139,140,141,142,143,144]
	CAR-T	Applied in clinic for hematological malignancies, is developing for solid tumors.	Various resistance and toxicities, application to solid tumors is difficult	Yes	Neuroblastoma, sarcoma, ovarian cancer, glioblastoma, breast cancer, colon cancer, mesothelioma, pancreatic carcinoma, liver cancer, et al.	[145,146,147,148,149,150,151]
Innate immunity	NK cell	Preclinical and clinical trials	Suppressive tumor microenvironment and limited contact frequency of NK cells with tumor cells	Yes	Liver cancer, glioblastoma, neuroblastoma, lung cancer, et al.	[152,153,154,155,156,157,158,159,160]
	Macrophage	Preclinical and clinical trials	Disturbed by tumor microenvironment	Yes	Liver cancer, colorectal cancer, pancreatic cancer, lung cancer, ovarian carcinoma, breast cancer, et al.	[161,162,163,164,165,166]
	Neutrophil	Preclinical and clinical trials	Lacking specificity and safety	Yes	Liver cancer, breast cancer, lung, et al.	[167,168,169]

## Abbreviations

Full name	Abbreviations
Hepatocellular carcinoma	HCC
Hepatitis C virus	HBV
Hepatitis B virus	HCV
HBV X protein	HBx
Tyrosine kinase inhibitors	TKIs
Chimeric antigen receptor	CAR
Food and drug administration	FDA
Tumor immune microenvironment	TIME
Biocompatible cationic polymers	BCPs
Overall survival	OS
Poly(ethylenimine)	PEI
Diethylene glycol diacrylate	DEGDA
*N*,*N*’-methylenebisacrylamide	MBA
*N*-carboxyanhydrides	NCAs
*1,1,1,3,3,3*-hexamethyldisilazane	HMDS
Epidermal growth factor receptor	EGFR
Poly (D,L-lactide-*co*-glycolide)	PLA
poly(beta-amino ester)s	PBAEs
Hyperbranched poly(kanamycin-MBA)	HPKM
Agar gel electrophoresis	AGE
Atomic force microscope	AFM
Double stranded RNA	dsRNA
Enhanced permeability and retention effect	EPR effect
Photoimmunotherapy	PIT
Antibody-polymer conjugates	ADCs
Ultrasound-targeted microbubble destruction	UTMD
Superparamagnetic iron oxide nanoparticles	SPIONs
Heat shock protein 70	HSP70
dendritic cell	DC
Major histocompatibility complex	MHC
Programmed cell death protein 1	PD-1
Programmed cell death ligand 1	PDL1
Cytotoxic T lymphocyte antigen-4	CTLA4
Tumor-associated antigens	TAAs
Natural killer	NK
Poly(ethylene glycol)-*block*-poly(lactide-co-glycolide)	PEG-PLGA
Tumor-associated macrophages	TAMs
Tumor-associated neutrophil	TAN
MYC gene inhibitor	MYCi
Regulatory T cells	Tregs
Adeno-associated virus	AAV
